# In vitro propagation of *Securidaca longipedunculata* (Fresen) from shoot tip: an endangered medicinal plant

**DOI:** 10.1186/s43141-019-0017-0

**Published:** 2020-01-20

**Authors:** Teklebrihan Lijalem, Tileye Feyissa

**Affiliations:** 1 Institute of Biotechnology, Addis Ababa University, P.O. Box 1176, Addis Ababa, Ethiopia; 2 Department of Microbial, Cellular and Molecular Biology, College of Natural and Computational Sciences, Addis Ababa University, P.O. Box 1176, Addis Ababa, Ethiopia

**Keywords:** Micropropagation, Plant growth regulators, *Securidaca longipedunculata*, Shoot initiation, Shoot multiplication

## Abstract

**Background:**

*Securidaca longipedunculata* Fresen is an indigenous medicinal plant in Africa that has an important place in both traditional and modern medicine. This plant is endangered because of high seed dormancy, low germination rate, and over exploitation. Therefore, micropropagation method is important to address these problems. The objective of this study is to develop a micropropagation protocol for *S. longipedunculata* from shoot tip explants.

**Results:**

Among different Clorox concentrations, seeds sterilized with 10% Clorox for 10 min resulted in 85% decontamination and 80% germination. Among different media used to evaluate the rate of seed germination, seeds that were de-coated and transversally cut at the tip and cultured on basal MS medium resulted in 100% germination. The highest percentage of shoot initiation (87%) was obtained on MS medium containing 1.0 mg/l 6-Benzylaminopurine (BAP). The highest mean shoot number per explant (8.5 ± 0.69) was achieved on MS multiplication medium containing 1.5 mg/l BAP in combination with 0.1 mg/l Indole-3-butyric acid (IBA). The highest mean number of roots per explant (3.73 ± 0.69) was obtained on MS medium containing 2.0 mg/l Indole-3-acetic-acid (IAA). Among plantlets transferred to greenhouse, 60% survived after acclimatization.

**Conclusions:**

This micropropagation protocol can be used for mass propagation of *S. longipedunculata* that contributes to its conservation and genetic improvement.

## Background

*Securidaca longipedunculata* Fresen is a small tree belonging to family Polygalaceae. It grows up to 12 m tall, with an often flattened or slightly fluted bole. It is an indigenous medicinal plant in Africa and plays an important role in both traditional and modern medicine. This tree is also commonly used as a pesticide against beetles in stored grains [1].

It is distributed in a wide range of climates ranging from subtropical, hot, and arid climate to summer rainfall and equatorial humid. It grows in different vegetation ranging from semi-arid scrub to dense forest, including bush habitats and gallery forests and woodland [2]. It is sensitive to frost and resistant to bush fires [3].

The root and bark are taken orally either powdered or as infusion for treating malaria, stomach problems, toothache, headache, sleeping sickness, cough, chest complaints, snakebite, and wound dressing and roots are used to kill seed storage pests. An infusion of the roots is used as a mouthwash in cases of toothache and is applied to cuts on the legs to treat inflammation. Powder from the burned roots is rubbed into small incisions made on the forehead to relieve headaches. Seeds are used for headache, fever, and rheumatism; leaves for snakebite, venereal diseases, and coughs, whereas the bark is used for stomach problems and as an arrow poison antidote [4].

*S. longipedunculata* has different chemical constituents including methylsalicylate, flavonoids, alkaloids elymoclavine, and dehydroelymoclavine, an ergoline compound and cinnamonic acid and the xanthones: 1,7-Dimethoxy-2-hydroxy-xanthone and 1, 4-dihydroxy-7-methoxy-xanthone [5]. A number of fatty acids and triglycerols such as coriolic acid, trans-9-dienoic acid, and 9-hydroxytetradeca-cis-5, trans-7-dienoic acid have been isolated from its seed oil [6].

The medicinal importance of *S. longipedunculata* has been recognized by the findings of different bioactive metabolites isolated from the bark yielding such as oleanolic acid, glycoside and alkaloid securiene used in treating convulsion in children, increased blood pressure and paralysis following infectious disease [7]. It is used for preparation of different medicines for neuromuscular blocking and negative inotropic and chronotropic cardiac effects [8]. Recent research findings showed *S. longipedunculata* induces apoptosis in brain tumor confirming its folkloric use in cancer management [9].

The alkaloid securinine confers activity against *Plasmodium falciparum* [10]. Some flavonoids that were isolated from this plant showed activity against many microorganisms and methanol extract of the root material against three major stored product pests [11, 12].

As the fruits usually stay on the tree for a year or more, it has been suggested that seeds should not be sown until it is 1-year old, but the seeds seem to lose viability quickly and germination is erratic. It is best to sow a seed soaked in cold water for 24 h in the soil and cover with grass and water until raining. It is difficult to cultivate largely because germination is poor and even after germination, seedling growth is slow and planting out is difficult because the taproot is broken easily [13]. *S. longipedunculata* is threatened because of the fact that roots are the target for people using this plant, which makes it difficult for the plant to survive constant harvesting. It suffers from uncontrolled harvesting for use in local medicines, as well as periodic droughts. It is considered vulnerable due to increasing threats to their habitats.

Little effort has also been made to develop propagation methods to facilitate planting of *S. longipedunculata* in agro-forestry systems. In vitro propagation of *S. longipedunculata* has considerable benefits for its availability as planting material and germplasm conservation. The protocol can facilitate mass propagation of superior genotypes for wider planting in agro-forestry systems or in its natural habitat where restoration and conservation is envisaged. This enables wise utilization of the plant through cultivation of elite plants for medicinal purposes in large quantities sustainably. In the present study, different germination rates of both coated and de-coated seeds of *S. longepedunculata* under different conditions were investigated, and in vitro propagation protocol was developed.

## Methods

### Plant materials and germination

#### Effect of Clorox concentrations on seed germination rate

Matured seeds were collected from Metema (Gendawuha), near the border with Sudan, about 1,000 km west of Addis Ababa, Amhara regional state, Ethiopia. The plant material was identified by the first author. Moreover, a voucher specimen of this material had been previously deposited in a publicly available National Herbarium. Seeds were washed under running tap water using detergent, washed in 70% ethanol for 2 min and rinsed three times with sterile double-distilled water followed by sterilization in 3%, 5%, 10%, and 15% sodium hypochlorite (Clorox) (Clorox, Oakland, California, USA) containing three drops of Tween-20 (Sigma-Aldrich, St. Louis, Missouri, USA) for 10 min. The seeds were then washed three times with sterile double-distilled water and cultured on basal MS [14] medium (Sigma-Aldrich, St. Louis, MI, USA). The number of germinated seeds was recorded.

#### Evaluation of germination rate

**Fig. 1:**
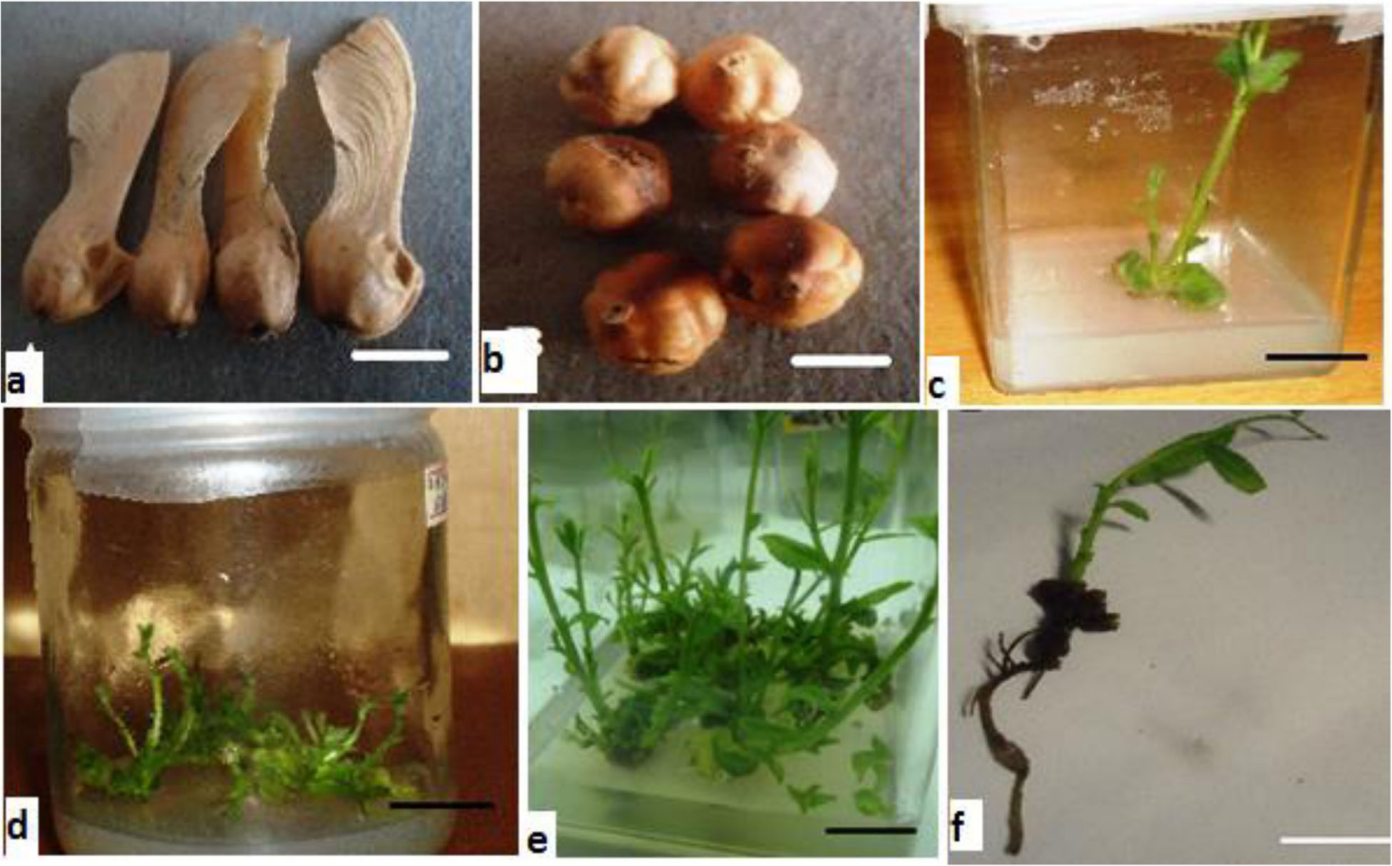
In vitro propagation of *S. longipedunculata.* **a** Seeds with coat. **b** De-coated seeds. **c** Seedling on growth regulator-free medium. **d** Shoot initiation on MS medium containing 1.0 mg/l BAP. **e** Shoot multiplication on MS medium containing 1.5 mg/l BAP in combination with 0.1 IBA. **f** Rooted shoots on MS medium containing 2.0 mg/l IAA. Bars represent 2 cm

Seeds were germinated in four conditions. (1) Both coated (Fig. 1a) and de-coated (Fig. 1b) seeds were sown on filter paper. (2) Both coated and de-coated seeds were sterilized and cultured on basal MS medium. (3) De-coated seeds with half-cut cotyledon were cultured on basal MS medium. (4) Coated seeds were planted in pots containing forest soil, sand, and compost in 2:1:1 ratio. Twenty seeds were used for each coated and de-coated experiments whereas in the experiments of coated seeds only, 40 seeds were used.

#### Sterilization of shoot explants

About 1.5 to 2.0-cm-long shoot explants were excised out from mother plants that were grown in pots in greenhouse. The shoots were washed under running tap water using detergent. The shoots were then surface sterilized using 70% ethanol (Dallul Pharmaceuticals, Addis Ababa, Ethiopia) for 30 s and rinsed three times with sterile double-distilled water followed by sterilization in 10% Clorox containing three drops of Tween-20 for 10 and 15 min. After sterilization, the explants were washed three times with sterile double-distilled water.

#### Shoot initiation

The shoots were trimmed to about 1.0 cm and cultured in baby food jars each containing 30 ml shoot initiation medium. The shoot initiation medium was MS medium containing 6-Benzylaminopurine (BAP) (0.5, 0.75, 1.0, 1.25, 1.5 mg/l) (Sigma-Aldrich, St. Louis, Missouri, USA) in combination with 0.5 mg/l thidiazuron (TDZ) or TDZ alone (0.5, 0.75, 1.0, 1.25, 1.5 mg/l) (Sigma-Aldrich, St. Louis, Missouri, USA), 30 g/l sucrose, 0.7% (w/v) agar (HiMedia, Mumbai, Maharashtra, India). The pH of the medium was adjusted to 5.8 before addition of agar. Basal MS medium was used as control. The cultures were maintained at a temperature of 25 ± 2 °C under light intensity of 20 μmol m^−2^ s^−1^ and 16 h photoperiod provided by cool-white fluorescent lamp (OSRAM GmbH, Munich, Germany). For each treatment, a total of 30 explants were used. There were six explants per jar with five replications. The experiment was repeated once. Number of dead and initiated shoots was recorded.

#### Shoot multiplication

Initiated shoots were cultured in Magenta GA-7 culture vessels (Sigma-Aldrich, St. Louis, Missouri, USA) containing 50-ml shoot multiplication medium. Shoot multiplication medium was MS medium containing different concentrations of BAP (0.5, 1.0, 1.5, 2.0, 2. 5 mg/l) in combination with Indole-3-butyric acid (IBA) (0.01, 0.1, 0.5 mg/l), TDZ (0.5, 1.0, 1.5, 2.0, 2. 5 mg/l) in combination with IBA (0.01, 0.1, 0.5 mg/l), or TDZ (0.5, 1.0, 1.5, 2.0, 2. 5 mg/l) in combination with α-Naphthalene acetic acid (NAA) (0.01, 0.1, 0.5 mg/l). Basal MS medium was used as control. The cultures were maintained at the same condition as for shoot initiation. For each treatment, a total of 30 explants were used. Number of shoots and shoot length were recorded after 4 weeks of culture. The whole experiment was repeated once.

#### Rooting

Rooting was done using full- and half-strength MS medium containing IBA (0.5, 1.0, 2.0, and 3.0 mg/l), Indole-3-acetic-acid (IAA) (0.5, 1.0, 2.0, and 3.0 mg/l), or NAA (0.5, 1.0, 2.0, and 3.0 mg/l). Basal MS medium was used as control. Shoots were maintained for a week in the dark and then transferred to the same condition as for shoot initiation for 3 weeks. Number of shoots that produced roots, number of roots per shoot, and length of roots were recorded after 5 weeks of culture.

#### Acclimatization

After 5 weeks in rooting medium, rooted shoots were removed from culture vessels and the roots were washed thoroughly under tap water and transferred to pots containing a mixture of soil, compost, and sand in a ratio of 2:1:1, respectively. Each pot was covered with a polyethylene bag and kept in a laboratory for 1 week before being transferred to glasshouse. The polyethylene bags were removed after a week of transferring to glasshouse. The plants were watered every day. The number of survived plants in the glasshouse was recorded after a month.

### Data analyses

Statistical analysis of quantitative data was carried out by SPSS computer software of version 20. A difference at probability level of *p* ≤ 0.05 was considered significant. Data were subjected to analysis of variance and variables that showed significant difference were compared by the LSD at 5% probability.

## Results

### Effect of Clorox concentrations on seed germination rate

The highest percentage of decontaminated seeds (90%) was obtained at 15% Clorox, but the germination percentage was reduced to 20% (Table 1). Among those Clorox concentrations, 10% Clorox concentration resulted in 85% decontaminated seeds and 80% seed germination. On the other hand, the least percentage of decontaminated seeds (15%) was obtained at 3% Clorox concentration.

**Table 1:** Effect of Clorox concentrations at 10 min exposure time on decontamination and germination rate of seeds of *S. longipedunculata*

Clorox concentration (%)	Decontaminated seeds (%)	Germinated seeds (%)
3	15	0
5	75	55
10	85	80
15	90	20

ANOVA result showed that Clorox concentrations significantly affected percentage of decontaminated seeds and germinated seeds (*p* ≤ 0.05). However, no significant difference was observed among 5%, 10%, and 15% Clorox concentrations with respect to seed decontamination, but the increasing concentration of Clorox to 15% negatively affected seed germination.

### Germination rate of seeds

Germination percentage was increased when the de-coated seeds were cut transversally. None of the coated seeds germinated in all media. The highest germination percentage (100%) of seeds was observed within 7–10 days on basal MS medium, when the seeds were de-coated and transversally cut at the tip. As shown in Table 2, 10% of the de-coated but not transversally cut seeds germinated under all tested conditions. The root part of the seedling showed faster growth than the aerial part in the first week of germination, but later the aerial part showed rapid growth and was elongated (Fig. 1c).

**Table 2:** Seed germination rate of *S. longipedunculata* under different growth conditions

Medium	Seeds	Germination time (day)	Germination rate (%)
Filter paper	Coated	-	0
	De-coated	90	10
MS medium	Coated	-	0
	De-coated	30	10
MS medium	De-coated and cut tip of cotyledon	7–10	100
Soil	Coated	-	0
	De-coated	90	10

### Shoot initiation

The use of a semi-solid MS medium containing BAP or in combination with TDZ or TDZ alone resulted in shoot initiation within a week. The highest percentage (87%) of shoot initiation was obtained on MS medium containing 1.0 mg/l BAP followed by 67% of shoot initiation on 1.0 mg/l BAP in combination with 0.5 mg/l TDZ (Table 3). The microshoots that were initiated on MS medium containing 1.0 mg/l BAP was higher in number per explant but shorter in length than the shoots obtained in 1.0 mg/l BAP in combination with 0.5 mg/l TDZ. The lowest percentage of shoot initiation (10%) was obtained on basal MS medium. In general, shoots were initiated better on MS medium containing BAP alone than MS medium containing TDZ alone or in combination with BAP.

**Table 3:** Effect of different concentrations of BAP in combination with TDZ on shoot initiation of *S. longipedunculata*

BAP (mg/l)	TDZ (mg/l)	Shoot initiation (%)
0.0	0.0	10
0.5	0.0	50
0.75	0.0	53
1.0	0.0	87
1.25	0.0	57
1.5	0.0	47
0.5	0.5	53
0.75	0.5	50
1.0	0.5	67
1.25	0.5	47
1.5	0.5	43
0.0	0.5	43
0.0	0.75	40
0.0	1.0	30
0.0	1.25	23
0.0	1.5	20

### Shoot multiplication

Shoot number was highly influenced by concentration and type of the growth regulators. Different combinations of BAP, IBA, TDZ, and NAA resulted in different responses. Different concentrations of BAP in combination with IBA produced better shoot number and length per explant than other growth regulator combinations (Figs. 1d and e). The highest shoot number per explant (8.50 ± 0.69) was obtained on medium containing 1.5 mg/l BAP in combination with 0.1 mg/l IBA. The lowest mean shoot number per explant (2.10 ± 0.12) was obtained on basal MS medium.

In terms of shoot length, the highest mean shoot length (2.11 ± 0.45 cm) was obtained on medium containing 2.5 mg/l BAP in combination with 0.1 mg/l IBA. However, there was no significant difference between shoots that were produced on 2.5 mg/l BAP in combination with 0.1 mg/l IBA and 2.5 mg/l BAP in combination with 0.01 mg/l IBA. The lowest mean shoot length (0.96 ± 0.40 cm) was recorded on MS medium containing 0.5 mg/l BAP in combination with 0.5 mg/l IBA (Table 4).

**Table 4:** Effect of different concentrations of BAP and IBA on shoot multiplication (shoot number and length) of *S. longipedunculata*

BAP (mg/l)	IBA (mg/l)	Shoot number per explant	Shoot length (cm)
0.0	0.0	2.10 ± 0.12^j^	1.22 ± 0.12^fh^
0.5	0.0	5.40 ± 0.54^efh^	1.58 ± 0.54^ce^
1.0	0.0	4.63 ± 0.24^gh^	1.52 ± 0.24^cd^
1.5	0.0	3.60 ± 0.22^hi^	1.11 ± 0.22^gh^
2.0	0.0	4.06 ± 0.37^h^	1.13 ± 0.37^gi^
2.5	0.0	7.13 ± 0.42^bce^	1.42 ± 0.42^de^
0.5	0.01	5.73 ± 0.30^dg^	1.84 ± 0.30^b^
0.5	0.1	5.80 ± 0.39^df^	1.51 ± 0.39^ce^
0.5	0.5	6.73 ± 0.40^cd^	0.96 ± 0.40^hi^
1.0	0.01	4.56 ± 0.26^gi^	1.10 ± 0.26^gi^
1.0	0.1	5.33 ± 0.32^efg^	1.31 ± 0.32^eg^
1.0	0.5	6.23 ± 0.37^dg^	1.18 ± 0.37^fh^
1.5	0.01	8.13 ± 0.53^ab^	1.33 ± 0.53^ef^
1.5	0.1	8.50 ± 0.69^a^	1.62 ± 0.69^c^
1.5	0.5	7.46 ± 0.38^ad^	1.01 ± 0.38^hi^
2.0	0.01	3.40 ± 0.15^i^	1.18 ± 0.15^fg^
2.0	0.1	6.30 ± 0.50^de^	1.22 ± 0.50^fh^
2.0	0.5	7.60 ± 0.56^ac^	1.99 ± 0.56^ab^
2.5	0.01	5.43 ± 0.28^efh^	2.08 ± 0.28^a^
2.5	0.1	5.76 ± 0.45^dg^	2.11 ± 0.45^a^
2.5	0.5	5.50 ± 0.32^efh^	1.24 ± 0.32^fh^

Means followed by the same superscript letters within a column are not significantly different at 5% probability level

In cases where TDZ alone or TDZ in combination with IBA was used, the highest mean number of shoots (6.20 ± 0.43) was produced on MS medium containing 0.5 mg/l TDZ alone followed by shoots cultured on MS medium containing 2.5 mg/l TDZ in combination with 0.5 mg/l IBA, which was 5.23 ± 0.49. The lowest mean shoot number (2.10 ± 0.12) was recorded on basal MS medium (Table 5).

**Table 5:** Effect of different concentrations of TDZ and IBA on *shoot multiplication (shoot number and length)* of *S. longipedunculata*

TDZ (mg/l)	IBA (mg/l)	Shoot number per explant	Shoot length (cm)
0.0	0.0	2.10 ± 0.12^l^	1.22 ± 0.03^b^
0.5	0.0	6.20 ± 0.43^a^	1.55 ± 0.09^a^
1.0	0.0	3.53 ± 0.18^efgi^	0.56 ± 0.03^f^
1.5	0.0	3.06 ± 0.13^gjk^	0.64 ± 0.04^e^
2.0	0.0	2.93 ± 0.11^ijk^	0.76 ± 0.04^de^
2.5	0.0	3.46 ± 0.19^efgj^	0.85 ± 0.06^d^
0.5	0.01	3.23 ± 0.15^fgj^	0.65 ± 0.04^ef^
0.5	0.1	2.86 ± 0.14^jk^	0.60 ± 0.05^f^
0.5	0.5	3.20 ± 0.20^fgjk^	0.42 ± 0.02^g^
1.0	0.01	3.60 ± 0.17^efgh^	0.59 ± 0.05^f^
1.0	0.1	3.66 ± 0.18^efgh^	0.39 ± 0.03^g^
1.0	0.5	2.56 ± 0.11^kl^	0.31 ± 0.02^g^
1.5	0.01	2.83 ± 0.11^jk^	0.75 ± 0.04^df^
1.5	0.1	3.16 ± 0.13^gjk^	0.98 ± 0.04^c^
1.5	0.5	3.03 ± 0.20^hijk^	0.42 ± 0.03^g^
2.0	0.01	3.70 ± 0.19^efg^	0.44 ± 0.03^g^
2.0	0.1	3.83 ± 0.27^ef^	0.32 ± 0.02^g^
2.0	0.5	4.50 ± 0.32^cd^	0.35 ± 0.02^g^
2.5	0.01	4.03 ± 0.23^de^	0.58 ± 0.04^f^
2.5	0.1	5.10 ± 0.36^bc^	0.65 ± 0.04^e^
2.5	0.5	5.23 ± 0.49^b^	0.76 ± 0.03^df^

Means followed by the same superscript letters within a column are not significantly different at 5% probability level

The highest mean shoot length (1.55 ± 0.09 cm) was obtained on MS medium containing 0.5 mg/l TDZ while the lowest mean shoot length (0.31 ± 0.02 cm) was produced on MS medium containing 1.0 mg/l TDZ in combination with 0.5 mg/l IBA.

In case of TDZ and NAA combinations, the highest mean shoot number per explant (4.70 ± 0.32) was obtained on MS medium containing 1.5 mg/l TDZ in combination with 0.5 mg/l NAA followed by MS medium containing 1.5 mg/l TDZ in combination with 0.1 mg/l NAA, which produced 4.56 ± 0.24 shoots per explant. However, there was no significant difference between mean shoot numbers per explant obtained on medium containing 1.5 mg/l TDZ in combination with 0.1 mg/l NAA and 0.5 mg/l TDZ in combination with 0.01 mg/l NAA. The lowest mean shoot number per explant (2.10 ± 0.12) was recorded on basal MS medium (Table 6).

**Table 6:** Effect of different concentrations of TDZ and NAA on *shoot multiplication (shoot number and length)* of *S. longipedunculata*

TDZ (mg/l)	NAA (mg/l)	Shoot number per explant	Shoot length (cm)
0.0	0.0	2.10 ± 0.12^f^	1.22 ± 0.03^a^
0.5	0.01	4.50 ± 0.34^ab^	0.41 ± 0.02^de^
0.5	0.1	3.60 ± 0.20^bc^	0.36 ± 0.02^eg^
0.5	0.5	3.13 ± 0.20^cd^	0.30 ± 0.02^fg^
1.0	0.01	2.50 ± 0.12^ef^	0.19 ± 0.01^hi^
1.0	0.1	2.96 ± 0.18^de^	0.43 ± 0.04^df^
1.0	0.5	2.56 ± 0.11^df^	0.34 ± 0.02^eg^
1.5	0.01	4.46 ± 0.24^ab^	0.68 ± 0.04^b^
1.5	0.1	4.56 ± 0.24^ab^	0.46 ± 0.02^d^
1.5	0.5	4.70 ± 0.32^a^	0.29 ± 0.01^g^
2.0	0.01	2.93 ± 0.21^df^	0.18 ± 0.01^hj^
2.0	0.1	4.06 ± 0.26^b^	0.57 ± 0.03^c^
2.0	0.5	3.66 ± 0.21^bc^	0.38 ± 0.03^ef^
2.5	0.01	2.70 ± 0.16^df^	0.16 ± 0.01^ij^
2.5	0.1	2.90 ± 0.14^df^	0.26 ± 0.02^gi^
2.5	0.5	3.26 ± 0.15^e^	0.25 ± 0.03^gh^

Means followed by the same superscript letters within a column are not significantly different at 5% probability level

TDZ in combination with NAA produced relatively shorter shoots. The highest mean shoot length (1.22 ± 0.03 cm) was obtained on growth regulator-free MS medium while the shortest mean shoot length (0.16 ± 0.01 cm) was produced on MS medium containing 2.5 mg/l TDZ in combination with 0.01 mg/l NAA (Table 6).

Most of the shoots produced on MS medium containing TDZ in combination with NAA were vitrified showing greenish-white color, formed calli like materials at the bottom end of the explants and were dwarf. The calli did not regenerate new microshoots when transferred to new fresh medium. TDZ in combination with NAA produced relatively lower mean shoot number and shorter shoots.

Among all growth regulators used in shoot multiplication treatments, the maximum mean shoot number per explant (8.5 ± 0.69) was obtained on MS medium containing 1.5 mg/l BAP in combination with 0.1 mg/l IBA. On the other hand, the lowest mean shoot number per explant (2.10 ± 0.12) was obtained on basal MS medium. MS medium containing 2.5 mg/l BAP in combination with 0.1 mg/l IBA resulted in the highest mean shoot length (2.11 ± 0.45 cm) while the shortest mean shoot length (0.16 ± 0.01 cm) was obtained on MS medium containing 2.5 mg/l TDZ in combination with 0.01 mg/l NAA.

### Rooting

Rooting was observed 4 weeks after culture of the shoots on root induction medium. However, most of the shoots developed roots by 5 weeks (Fig. 1f). The highest mean root number per shoot (3.73 ± 0.69) was obtained on full-strength MS medium containing 2.0 mg/l IAA. On the other hand, none of the shoots induced root on basal MS medium (Table 7). Although the growth regulators had significant effect on root induction, there was no significant difference among roots induced on full-strength MS medium containing 0.5, 1.0, or 2.0 mg/l IAA or 3.0 mg/l NAA, which showed relatively better root induction among other treatments.

**Table 7:** Effect of MS salt strength and different auxin concentrations on number of roots per shoot and root length of *S. longipedunculata*

Salt strength	IAA (mg/l)	NAA (mg/l)	IBA (mg/l)	Root number per shoot	Root length (cm)
Full strength	0	0	0	0.0 ± 0^g^	0.0 ± 0^h^
	0.5	0	0	3.53 ± 0.44^a^	0.21 ± 0.17^bde^
	1	0	0	3.06 ± 0.33^ab^	0.19 ± 0.01^cd^
	2	0	0	3.73 ± 0.69^a^	0.26 ± 0.02^b^
	3	0	0	3.10 ± 0.33^ab^	0.34 ± 0.02^a^
	0	0.5	0	0.83 ± 0.13^f^	0.08 ± 0.01^f^
	0	1	0	3.56 ± 0.58^a^	0.33 ± 0.04^a^
	0	2	0	2.10 ± 0.25^be^	0.15 ± 0.01^def^
	0	3	0	1.46 ± 0.36^e^	0.12 ± 0.02^eg^
	0	0	0.5	0.76 ± 0.13^f^	0.12 ± 0.02^eg^
	0	0	1	1.33 ± 0.21^e^	0.22 ± 0.03^bc^
	0	0	2	1.13 ± 0.19^e^	0.18 ± 0.03^ce^
	0	0	3	1.76 ± 0.26^cde^	0.23 ± 0.03^bde^
Half strength	0.5	0	0	2.43 ± 0.22^bd^	0.33 ± 0.03^a^
	1	0	0	2.30 ± 0.16^be^	0.17 ± 0.01^cf^
	2	0	0	2.66 ± 0.34^b^	0.19 ± 0.01^cf^
	3	0	0	2.36 ± 0.23^be^	0.26 ± 0.02^b^
	0	0.5	0	0.50 ± 0.09^fg^	0.07 ± 0.01^g^
	0	1	0	2.50 ± 0.29^bc^	0.26 ± 0.03^bd^
	0	2	0	1.73 ± 0.16^cdf^	0.12 ± 0.01^eg^
	0	3	0	0.83 ± 0.11^f^	0.09 ± 0.01^fg^
	0	0	0.5	0.50 ± 0.09^f^	0.07 ± 0.01^g^
	0	0	1	0.96 ± 0.13^ef^	0.14 ± 0.02^de^
	0	0	2	0.73 ± 0.16^f^	0.09 ± 0.02^fg^
	0	0	3	1.20 ± 0.06^e^	0.12 ± 0.01^eg^

Means followed by the same superscript letters within a column are not significantly different at 5% probability level

The highest mean root length (0.34 ± 0.02 cm) was observed on full-strength MS medium containing 3.0 mg/l IAA. However, no significant difference was observed among roots developed on full-strength MS medium containing 3.0 mg/l IAA and half-strength MS medium containing 0.5 mg/l IAA. Among all concentrations, the lowest mean root length (0.07 ± 0.01 cm) was observed on half-strength MS medium containing 0.5 mg/l NAA or 0.5 mg/l IBA.

Full-strength MS medium containing different concentrations of IAA (0.5 and 1.0 mg/l), NAA (1.0 and 2.0 mg/l), and IBA (2.0 and 3.0 mg/l) produced enlarged callus-like structure on and around the root tip. This structure had an influence on reducing the number and length of roots compared to treatments without callus-like growth.

### Acclimatization

Out of 102 plantlets which were planted in pots containing red soil, compost, and sand (2:1:1) placed in a green house, 60% survived after a month. Most of the dead plants were due to small root number or hairs, fungal contamination, and rotting. None of the plants survived in pots which were not covered by polyethylene bags and those containing unsterilized soil, compost, and sand.

## Discussion

The result showed that increasing Clorox concentration increases decontamination, but decreases viability of the seeds. The percentage of commercial Clorox needs to be varied depending on the species and environment [15]. On the other hand, the work is in disagreement with that of Srivastava et al. [16] who found that sterilization with NaOCl did not give acceptable sterilization even on increasing concentration over *Aconitum heterphyllum* (medicinal herb) seeds. According to our results, the highest percentage of decontaminated seeds was observed at 15% sodium hypochlorite (Clorox), but low germination percentage. Guanih et al. [15] found that sterilization of de-coated seeds of a medicinal plant, *Dryobalanops lanceolata* with 30% Clorox concentration for 5 min and cultured on MS medium showed less than 20% contamination, but sterilization of coated seeds with 50% Clorox concentration for more than 20 min broke the seed coat and reduced seed viability.

Among those Clorox concentrations, 10% Clorox resulted in 85% decontaminated seeds and the highest germination percentage (80%). Talei et al. [17] reported on medicinal herb, *Andrographis pancuatanee,* absence of significant correlation between contamination and germination percentages. Ghareeb and Taha [18] obtained 100% of decontamination for cultures and survival of *Antigonon leptopus* explants using 10% Clorox for 3 min. In general, the lower the Clorox concentration, the higher the contamination and germination percentage.

Our result showed that the de-coated seeds showed better germination than coated seeds. As Zulu et al. [19] reported that removing the seed coat could reduce contamination sources while improving the imbibitions of water and subsequent germination in *S. longipedunculata*. In addition, Negash [20] reported successful germination (91 ± 2.7%) of de-coated seeds of a tree plant, *Prunus africana*, in a laboratory.

In the present study, the germination on full-strength MS medium was best among other half-strength germination media. According to Pandey et al. [21], the highest percentage of seed germination (95%) from an important medicinal plant, *Psoralea corylifolia,* was recorded on full-strength MS medium. Maślanka and Bach [22] obtained higher percentage of germinating seeds on half-strength than full-strength MS medium after 6 months of culture. However, after 10 months, the highest germination percentage was observed on full-strength MS medium. Our result showed that none of the coated seeds germinated in all media. The reason may be due to the presence of the seed coat hampered the imbibitions of water. Pandey et al. [21] suggested the presence of seed coat challenges the entry of water and oxygen into the embryo. In contrast, Zulu et al. [19] obtained 45% germination rate of intact seeds of *S. longipedunculata* using GA_3_.

Among all media, the highest germination percentage (100%) of seeds was observed within 7–10 days on MS medium, when the seeds were de-coated and transversally cut at the tip. This is partly due to the removing of the seed coat facilitated the uptake of water and cutting at the tip reduces the size to use the limited amount of moisture on the MS medium. Similarly, Ganaie et al. [23] reported maximum germination percentage (96.66%) of *Arnebia benthamii* by removing the seed coat within mean germination time of around 4 days.

In this study, the effect of plant growth regulators on shoot induction from shoot explants was investigated. Sixteen different treatments including the control were used to induce shoots from *S. longipedunculata* shoot explants. These treatments, with various combinations of BAP and TDZ resulted in shoot initiation. The percentage response of explants for shoot induction, shoot number, and shoot length varies according to the type and concentration of cytokinins used [24]. The response of plants to different types and concentrations of growth regulators varies because of differences in endogenous level of growth regulators. In the present study, the shoots induced microshoots within a week on full-strength MS medium.

The highest percentage (87%) of explants survived on MS medium containing 1.0 mg/l BAP. Tiwari et al. [25] reported that MS medium containing 1.0 mg/l BAP was better for establishment of *Trichosanthes dioica* from nodal explants. The microshoots that resulted from MS medium containing 1.0 mg/l BAP were higher in number, but shorter in length than the shoots obtained from 1.0 mg/l BAP in combination with 0.5 mg/l TDZ. Ahmed and Anis [26] reported exposure of culture to TDZ had an adverse effect.

In general, the shoots were initiated better on MS medium containing BAP alone than MS medium containing TDZ alone or in combination with BAP. According to Kumar and Singh [27], BAP was found to perform better in shoot initiation of the multipurpose desert tree, *Prosopis cineraria*. Similarly, Malik and Saxena [28] pointed out the advantageous outcome of BAP for shoot induction as they observed on tissue culture of grain legume, *Phaseolus vulgaris.*

Application of BAP in combination with IBA with different concentrations induced the highest shoot number and length per explant. From our result, the MS medium containing 1.5 mg/l BAP in combination with 0.1 mg/l IBA produced the highest mean number of shoots (8.50 ± 0.69). Medium supplemented with auxin at low concentrations in combination with cytokinin promote the growth and formation of new shoots, consequently, increasing multiplication rate [29]. Adsul et al. [30] reported that nodal buds cultured on MS medium supplemented with 2.0 mg/l BAP in combination with 0.5 mg/l IBA resulted in production of maximum number of shoots (17.1 ± 1.2) of *Ceropegia mohanramii*. In contrast, Fraternale et al. [31] reported high concentration of IBA with BAP (1.5 mg/l) in MS medium was suitable for shoot multiplication of *Bupleurum fruticosum*. According to Askari-Khorasgani et al. [32], in comparison to the application of BAP alone, the combination of BAP along with IBA, led to more shoot formation. In addition, Ahmad et al. [33] stated that the response of the explants is a communal result of endogenous and exogenous plant growth regulators concentrations. Inclusion of a low concentration of auxin along with cytokinin activates much higher rate of shoot multiplication [34, 35]. Bramhanapalli et al. [36] reported high frequency shoot bud induction from different explants on MS medium containing 2.0 mg/l BAP as compared to other tested media indicating the high influence of BAP on shoot multiplication.

The highest mean shoot length (2.11 ± 0.45 cm) was obtained on 2.5 mg/l BAP in combination with 0.1 mg/l IBA whereas the lowest shoot length (0.96 ± 0.40 cm) was recorded on MS medium containing 0.5 mg/l BAP in combination with 0.5 mg/l IBA. This is surprising as the concentration of BAP increased, shorter shoots are expected because of apical dominance indicating the present finding requires further investigation.

On medium containing TDZ alone or in combinations with IBA, the highest mean number of shoots per explant (6.20 ± 0.43) was produced on MS medium containing 0.5 mg/l TDZ alone. Mirici [37] reported that TDZ alone prompted reasonable shoot multiplication in *Astragalus polemoniacus*.

In terms of length, the highest mean shoot length (1.55 ± 0.09 cm) was obtained on MS medium containing 0.5 mg/l TDZ. Aasim et al. [38] showed that the maximum number of shoots was recorded on MS medium containing 0.4 mg/l TDZ with highest shoot length of 1.20 cm. On the other hand, the shortest mean shoot length (0.31 ± 0.02 cm) was produced on MS medium containing 1.0 mg/l TDZ in combination with 0.5 mg/l IBA. Bisht et al. [39] who worked on *Polygonatum verticillatum*, suggested that the promoter effect of TDZ regarding growth is due to its own biological activities similar to N-substituted cytokinin or it may induce the synthesis or accumulation of an endogenous cytokinin. In case of TDZ and NAA combinations, the highest mean number of shoots (4.70 ± 0.32) was produced on MS medium containing 1.5 mg/l TDZ in combination with 0.5 mg/l NAA

Generally, among all growth regulators used in shoot multiplication, the highest mean shoot number (8.5 ± 0.69) was obtained on MS medium containing 1.5 mg/l BAP in combination with 0.1 mg/l IBA. On the other hand, the lowest mean shoot number per explant (2.10 ± 0.12) was obtained on basal MS medium. MS medium containing 2.5 mg/l BAP in combination with 0.1 mg/l IBA produced the highest mean shoot length (2.11 ± 0.45 cm) while the shortest mean shoot length (0.16 ± 0.01 cm) was produced on MS medium containing 2.5 mg/l TDZ in combination with 0.01 mg/l NAA and this short shoot production could be due to higher inhibition of apical dominance by TDZ than BAP.

Interestingly, full-strength MS medium containing 2.0 mg/l IAA, resulted in better rooting response (3.73 ± 0.69) as compared to IBA and NAA contrary to the most common half-strength MS medium used for rooting. IAA was found to be effective on rooting of *Malus pumila* [40]. In addition, Alagesaboopathi [41] reported 55.14% of rooting from *Andrographis macrobotrys* using IAA. Contrary to the results of our study, Shen et al. [42] pointed out that IAA failed to induce root formation on a medicinal tree plant, *Casuarina cunninghamiana.* It is important to work more on in vitro production of root and also root culture as the roots are used for medicinal purposes.

In this study, 60% plants survived in glasshouse after a month. This low survival percentage might be due to small root number or hairs, fungal contamination, and rotting. According to Bohidar et al. [43] who worked on medicinal plant, *Ruta graveolens,* the dead plantlets were due to improper development of root system in the culture. None of the plantlets survived in pots which were not covered with polyethylene bags.

## Conclusions

*Securidaca longipedunculata* is being destroyed because its root is used for medicinal purpose. Its natural regeneration is very difficult as the seed germination rate of the plant is very low. There is a need to produce superior genotypes of this plant to use its root for medicinal purposes and saving the natural stand. Therefore, the present study is very important for mass propagation of superior genotypes and conservation of this very important medicinal plant.

## Data Availability

Not applicable

## Abbreviations

BAP: 6-Benzylaminopurine
IAA: Indole-3-acetic-acid
IBA: Indole-3-butyric acid
MS: Murashige and Skoog (1962)
NAA: α-Naphthalene acetic acid
TDZ: Thidiazuron

## References

[1] Ojewole JAO (2008). Analgesic, anti-inflammatory and hypoglycaemic effects of *Securidaca longepedunculata* (Fresen.) [Polygalaceae] root bark aqueous extract. Inflammopharmacology.

[2] Dery BB, Otsyina R, Ng’atigwa C (1999). Indigenous knowledge of medicinal trees and setting priorities for their domestication in Shinyanga region, Tanzania.

[3] Beentje HJ (1994). Kenya trees, shrubs and lianas.

[4] Orwa C, Mutua A, Kindt R, Jamnadass R, Anthony S (2009). Agroforestree Database: a tree reference and selection guide version 4.0.

[5] Rakuambo NC, Meyer JJ, Hussein A (2004). Xanthone isolated from *Securidaca longepedunculata* with activity against erectile dysfunction. Fitoterapia.

[6] Smith CR, Madrigal RV, Plattner RD (1979). New conjugated hydroxydienoic fatty acids and acetotriacylglycerols from *Securidaca longipedunculata* seed oil. Biochim Biophys Acta.

[7] Harborne JB, Baxter H, Taylor F (1993). Pyrrolizidine alkaloids. Phytochemical dictionary, Bristol.

[8] Ojewole JAO, Ilesanmi ORS, Olayiwola G (2001). Antibronchoconstrictor effects of *Securidaca longepedunculata* (Fresen.) root bark methanolic extract in guinea-pigs. Acta Med Biol.

[9] Ngulde SI, Sandabe UK, Abounader R, Dawson TK, Zhang Y, Iliya I, Hussaini IM (2019). Ethanol extract of *Securidaca longipedunculata* induces apoptosis in brain tumor (U87) cells. Biomed Res Int Vol.

[10] Maiga A, Diallo D, Fane S, Sanogo R, Paulsen BS, Cisse B (2005). A survey of toxic plants on the market in the district of Bamako, Mali: traditional knowledge compared with a literature search of modern pharmacology and toxicology. J Ethnopharmacol.

[11] Ajali U, Chukwurah B (2004). Antimicrobial activity of *Securidaca longepedunculata*. Phytomedicine.

[12] Adejuwon AO, Adeosun AM, Tsygankova VA, Falaseao OFO, Amusa FI (2019). Phytochemical screening and antimicrobial efficacy of the root bark of *Securidaca longipedunculata* extracts. Am J Res Med Sci.

[13] Mbuya LP, Msanga CK, Ruffo CK, Birnie A, Tengas B (1994). Useful trees and shrubs for Tanzania: identification, propagation and management for agricultural and pastoral communities.

[14] Murashige T, Skoog F (1962). A revised medium for rapid growth and bioassays with tobacco cell cultures. Physiol Plant.

[15] Guanih VS, Mahali A, Tuyok M (2004). Seed sterilization of *Dryobalanops lanceolata* Burck. Sepilok Bulletin.

[16] Srivastava N, Kamal B, Sharma V, Negi YK, Dobriyal AK, Gupta S, Jadon VS (2010). Standardization of sterilization protocol for micropropagation of *Aconitum heterophyllum*, an endangered medicinal herb. Academ Arena.

[17] Talei D, Saad MS, Yusop MK, Abdulkadir M, Valdiani A (2011). Effect of different surface sterilizers on seed germination and contamination of king of bitters (*Androgrphis paniculata* Nees). Am Eurasian J Agric Environ Sci.

[18] Ghareeb ZF, Taha LS (2018). Micropropagation protocol for *Antigonon leptopus* an important ornamental and medicinal plant. J Genet Eng Biotechnol.

[19] Zulu D, Blackson L, Thokozani K, Gudeta W, Teklehaimanot Z, Dominic SB, Sarasan GV, Stevenson PC (2011) Propagation of the African medicinal and pesticidal plant, Securidaca longepedunculata. Afr J Biot 10:5988-92

[20] Negash Legesse (2004). Rapid seed-based propagation method for the threatened African cherry (Prunus africana). New Forests.

[21] Pandey P, Mehta M, Upadhyay R (2014) Effect of different media, photoperiod in seeds germination and effects of plant growth regulators on seedling growth of endangered medicinal plant *Psoralea corylifolia* Linn. Am J Ther Phytomedicine 2:102–109

[22] Maślanka M, Bach A (2014). Induction of bulb organogenesis in *in vitro* cultures of tarda tulip (*Tulipa tarda* Stapf.) from seed-derived explants. In Vitro Cell Dev Biol-Plant.

[23] Ganaie AK, Aslam S, Nawchoo IA (2011). No chilling obligation forgermination in seeds of *Arnebia benthamii*: a critically endangered alpine medicinal plant of north-west Himalayas. Int J Biodivers Conserv.

[24] Verma M, Bansal YK (2014). Effect of a potent cytokinin thidiazuron (TDZ) on i*n-vitro* regeneration of *Hedychium coronarium*: a valuable medicinal plant. Int J Rec Biot.

[25] Tiwari AK, Singh D, Tripathi S, Mishra N, Singh RB (2010). Effect of BAP and kinetin on shoot initiation of *Trichosanthes dioica* Roxb: An important medicinal plant. J Med Plants.

[26] Ahmed MR, Anis M (2012). Role of TDZ in the quick regeneration of multiple shoots from nodal explant of *Vitex trifolia* L. an important medicinal plant. Appl Biochem Biotecnol..

[27] Kumar S, Singh N (2010). Micropropagation of *Prosopis cineraria* (L.) Druce.: a multipurpose desert tree. Rep Opinion.

[28] Malik KA, Saxena PK (1992). Regeneration in *Phaseolus vulgaris* L.: high frequency induction of direct shoot formation in intact seedlings by N6-benzylaminopurine and thidiazuron. Planta.

[29] Pierik RLM (1997). *In vitro* culture of higher plants.

[30] Adsul AA, Chavan JJ, Gaikwad NB, Gurav RV, Dixit GB, Yadav SR (2019). *In vitro* regeneration approaches for restoration of *Ceropegia mohanramii*—an endemic and critically endangered asclepiad. J Genet Eng Biotechnol.

[31] Fraternale D, Giamperi L, Ricci D, Rocchi MBL (2002). Micropropagation of *Bupleurum fruticosum*: the effect of triacontanol. Plant Cell Tiss Org Cult.

[32] Askari-Khorasgani O, Mortazaeinezhad F, Otroshy M, Golparvar AR, Moeini A (2013). Regeneration of an endangered medicinal plant *Kelussia odoratissima*. Int J Agri Crop Sci.

[33] Ahmad N, Faisal M, Anis M, Aref IM (2010). *In-vitro* callus induction and plant regeneration from leaf explants of *Ruta graveolens* L. S Afr J Bot.

[34] Tsay HS, Gau TG, Chen CC (1989). Rapid clonal propagation of *Pinellia ternata* by tissue culture. Plant Cell Rep.

[35] Rout GR (2004). Effect of cytokinins and auxins on micropropagation of *Clitorea ternatea* L. Biol Lett.

[36] Bramhanapalli M, Thogatabalija L, Gudipalli P (2017). Efficient *in-vitro* plant regeneration from seedling-derived explants and genetic stability analysis of regenerated plants of *Simarouba glauca* DC. by RAPD and ISSR markers. In Vitro Cell Dev Biol-Plant.

[37] Mirici S (2004). High frequency of adventitious shoot regeneration from leaf and leaf petiol ofendemic *Astragalus polemoniacus* Bunge. Selçuk University Agricultural Faculty Publications.

[38] Aasim M, Khawar KM, Sancak CS, Ozcan S (2009). *In-vitro* shoot regeneration of fenugreek (*Trigonella foenumgraceum* L.). Am.-Eurasian J Sustain Agric.

[39] Bisht S, Snehlata B, Bahndari S (2011). *In-vitro* micropropagation in *Polygonatum verticillatum* (L): an important threatened medicinal herb of Northern India. Physiol Mol Biol Plants.

[40] Alvarez R, Nissen S, Sutter EG (1989). Relationship between Indole-3-Acetic Acid Levels in Apple (*Malus pumila* Mill) rootstocks cultured *in-vitro* and adventitious root formation in the presence of Indole-3-Butyric Acid. J Plant Growth Regul.

[41] Alagesaboopathi C (2012). Effect of IAA and IBA on the rooting of *Andrographis macrobotrys* Nees stem cuttings: an endangered medicinal plant of India. Int J Recent Sci Res.

[42] Shen X, Castle WS, Gmitter FG (2010). *In-vitro* shoot proliferation and root induction of shoot tip explants from mature male plants of *Casuarina cunninghamiana* Miq. Hort Sci.

[43] Bohidar S, Thirunavoukkrasu M, Rao TV (2008). Effect of plant growth regulators on *in-vitro* micropropagation of garden rue (*Ruta graveolens* L.). Int J Integr Biol.

